# Herbal medicine use among urban residents in Lagos, Nigeria

**DOI:** 10.1186/1472-6882-11-117

**Published:** 2011-11-25

**Authors:** Ibrahim Adekunle Oreagba, Kazeem Adeola Oshikoya, Mercy Amachree

**Affiliations:** 1 Pharmacology Department, College of Medicine, University of Lagos, P.M.B 12003 Idiaraba, Lagos, Nigeria; 2 Pharmacology Department, Lagos State University College of Medicine, Ikeja, Lagos, Nigeria; 3 Academic Division of Child Health, Medical School in Derby, University of Nottingham, Royal Derby Hospital, Uttoxeter Road, Derby, DE22 3DT, UK

## Abstract

**Background:**

Over three-quarter of the world's population is using herbal medicines with an increasing trend globally. Herbal medicines may be beneficial but are not completely harmless.

This study aimed to assess the extent of use and the general knowledge of the benefits and safety of herbal medicines among urban residents in Lagos, Nigeria.

**Methods:**

The study involved 388 participants recruited by cluster and random sampling techniques. Participants were interviewed with a structured open- and close-ended questionnaire.

The information obtained comprises the demography and types of herbal medicines used by the respondents; indications for their use; the sources, benefits and adverse effects of the herbal medicines they used.

**Results:**

A total of 12 herbal medicines (crude or refined) were used by the respondents, either alone or in combination with other herbal medicines. Herbal medicines were reportedly used by 259 (66.8%) respondents. 'Agbo jedi-jedi' (35%) was the most frequently used herbal medicine preparation, followed by 'agbo-iba' (27.5%) and Oroki herbal mixture^® ^(9%). Family and friends had a marked influence on 78.4% of the respondents who used herbal medicine preparations. Herbal medicines were considered safe by half of the respondents despite 20.8% of those who experienced mild to moderate adverse effects.

**Conclusions:**

Herbal medicine is popular among the respondents but they appear to be ignorant of its potential toxicities. It may be necessary to evaluate the safety, efficacy and quality of herbal medicines and their products through randomised clinical trial studies. Public enlightenment programme about safe use of herbal medicines may be necessary as a means of minimizing the potential adverse effects.

## Background

Herbal medicine is an integral part of "traditional medicine" (TM). TM has a broad range of characteristics and elements which earned it the working definition from the World Health Organization (WHO). Traditional medicines are diverse health practices, approaches, knowledge and beliefs that incorporate plant, animal and/or mineral based medicines, spiritual therapies, manual techniques and exercises which are applied singularly or in combination to maintain well-being, as well as to treat, diagnose or prevent illness [1]. In the developed countries, TM has been adapted outside its indigenous culture to "Complementary" or "Alternative" medicine (CAM) [2].

Globally, people developed unique indigenous healing traditions adapted and defined by their culture, beliefs and environment, which satisfied the health needs of their communities over centuries [3]. The increasing widespread use of TM has prompted the WHO to promote the integration of TM and CAM into the national health care systems of some countries and to encourage the development of national policy and regulations as essential indicators of the level of integration of such medicine within a national health care system [3,4].

Herbal medicines, also called botanical medicines or phytomedicines, refer to herbs, herbal materials, herbal preparations, and finished herbal products that contain parts of plants or other plant materials as active ingredients [1]. The plant materials include seeds, berries, roots, leaves, bark or flowers [5]. Many drugs used in conventional medicine were originally derived from plants. Salicylic acid is a precursor of aspirin that was originally derived from white willow bark and the meadowsweet plant (*Filipendula ulmaria *(L.) Maxim.) [6]. Quinine and Artemesinin are antimalarial drugs derived from *Cinchona pubescens *Vahl bark and *Artemisia annua *L. plant, respectively [7,8]. Vincristine is an anticancer drug derived from periwinkle (*Cantharnthus rosues *Linn. G. Donn.) [9]. Morphine, codeine, and paregoric, derived from the opium poppy (*Papaver somniferum *L.), are used in the treatment of diarrhea and pain relief [10]. Digitalis is a cardiac glycoside derived from foxglove plant (*Digitalis purpurea *L.); an herb in use since 1775 [11].

In folklore medicine in Nigeria *Rauwolfia vomitoria *(Afzel) is used for treating hypertension, stroke, insomnia and convulsion [12] and *Ocimum gratissimum *L. is used for treating diarrheal diseases [13]. the seeds of *Citrus parasidi* Macfad. are effective in treating urinary tract infections that are resistant to the conventional antibiotics [14]; pure honey healed infected wounds faster than eusol [15]; dried seeds of *Carica papaya *L. is effective in the treatment of intestinal parasitosis [16]; the analgesic and inflammatory effects of *Garcinia kola *Heckel is known to enhance its use for osteoarthritis treatment [17]; and *Aloe vera *Mill. gel is as effective as benzyl benzoate in the treatment of scabies [18]. Similarly, in South Africa, plant extracts with muscle relaxant properties are used by traditional birth attendants (TBAs) to assist in child deliveries [19].

Over 80% of the populations in some Asian and African countries depend on traditional medicine for primary health care [1]. The WHO estimates that in many developed countries, 70% to 80% of the population has used some form of alternative or complementary medicine including Ayurvedic, homeopathic, naturopathic, traditional oriental, and Native American Indian medicine [2]. It is also recognised by the WHO that herbal medicines are the most popular form of traditional medicine, and are highly lucrative in the international medicine market. Annual revenues in Western Europe were estimated as US$ 5 billion in 2003-2004, in China the revenue was estimated as US$ 14 billion in 2005, and in Brazil it was US$ 160 million in 2007 [1].

Despite the widespread use of herbal medicines globally and their reported benefits, they are not completely harmless. The indiscriminate, irresponsible or non-regulated use of several herbal medicines may put the health of their users at risk of toxicity [20-23]. Also, there is limited scientific evidence from studies done to evaluate the safety and effectiveness of traditional medicine products and practices [1]. Adverse reactions have been reported to herbal medicines when used alone [24] or concurrently with conventional or orthodox medicines [25]. Despite the international diversity and adoption of TM in different cultures and regions, there is no parallel advance in international standards and methods for its evaluation [1,2]. National policies and regulations also are lacking for TM in many countries and where these are available; it is difficult to fully regulate TM products, practices and practitioners due to variations in definitions and categorizations of TM therapies [3]. Lack of knowledge of how to sustain and preserve the plant populations and how to use them for medicinal purposes is a potential threat to TM sustenance [1,2].

Previous studies of herbal medicine use in Nigeria were focused on adults with various forms of chronic illnesses [26-28], pregnant women [29] and children with chronic illnesses [30]. The use of herbal medicines among a general population without chronic health conditions has never been evaluated in Nigeria or other African countries. This study was therefore aimed at assessing the extent of use and the general knowledge of the benefits and safety of herbal medicines among residents in Surulere Local Government Area (LGA) in Lagos, Nigeria.

## Methods

This is a descriptive study involving the residents of Surulere LGA in Lagos. Lagos is the smallest but most populous state in Nigeria, with an area of 75, 755 hectares. As of 2006 national census, the population of Lagos State was 15 million. About 1.7 million (5%) of these inhabitants live in Surulere LGA. The choice of Surulere LGA was informed by its large population size and heterogeneity. Surulere LGA is partly residential, industrial and commercial.

The study involved 388 participants recruited by cluster and random sampling techniques. The sample size was calculated using the formula: N = Z^2^Pq/d^2^, where N = minimum sample size, Z = confidence level at 95%, P = prevalence of herbal medicine use from previous studies in Nigeria, q = (1-P), and d = level of precision [31].

### Ethical Consideration

This study did not have the approval by an ethics committee; however it was evaluated and approved by the Medical Officer of Health of Surulere Local Government Authority. A structured interview-administered questionnaire was the instrument used for the study. The questionnaire was translated into Pidgin English and Yoruba Language by a Language specialist and was pre-tested with a sample group of 20 participants who were residents in another Local Government Area in Lagos. Modifications were made to the questionnaire after translating the results of the pilot study from Pidgin English and Yoruba Language back into English Language by the same Language specialist, by so doing, we eliminated any bias.

The participants were given written information, in English or Yoruba Language, or Pidgin English, to read prior to taking part in the study. They were informed that accepting to participate in the study is taken as consent from them. The participants were assured utmost confidentiality of the information tendered during the interview.

### The Questionnaire

The questionnaire was developed from previous studies on CAM and herbal medicine use in paediatric and adult patients in Nigeria [26-30,32,33]. It was used to obtain the following information: demographics of the participants; history of past and present use of herbal medicine, and the types used. Information was also obtained on the sources, benefits and adverse effects of the herbal medicines used. The questions asked were both open- and close-ended. The open-ended questions were focused on the types, sources, benefits and adverse effects of the herbal medicines used by the participants. The section also allowed the participants to give multiple responses to the open-ended questions. In this study, herbal medicine is considered as the use of phytomedicine according to the definition of the World Health Organization [1].

Participants who had used herbal medicine at least once in the last six months were regarded as herbal medicine users; those who had never used it at all were considered non-users; and those who had used herbal medicine at least once in their lifetime but not during the previous six months were considered herbal medicine-exposed [30].

### Participant recruitment

Surulere LGA was divided into ten clusters, each cluster representing a ward. Forty adult (18 years and above) participants were selected randomly from each household in a ward.

Participants suffering from any form of chronic illness such as hypertension, asthma, diabetes mellitus, cancer, arthritis, HIV/AIDS, epilepsy, or sickle cell anaemia were excluded. Altogether, 400 participants were selected for the study but only 388 (97%) of them voluntarily participated.

### Structured interview of the participants

The participants were interviewed in their respective homes. Each participant was interviewed by either one of the researchers (MA) or research assistants (specifically employed and trained for the study), after the contents of the questionnaire had been explained to them in their native language (illiterate participants) or English (literate participants). The structured interview adopted in this study allowed us to explain each of the terminologies used in the questionnaire to the participants. Also, the method enabled us to eliminate biases that characterized previous studies involving self-administered questionnaires [27-29,32,33]. Such limitations include the use of terms and concepts that are confusing to participants and poorer response rates from incomplete filling of the questionnaire. Our method of interview also eliminated incomplete filling-in of the questionnaires by participants.

### Identification of components of the herbal medicines

Information about the components of the crude herbal medicines was obtained during the interview from the respondents who had used or were still using crude herbal medicines. For the respondents who had used or still using herbal medicines refined into their packaged forms, the trade names of the herbal products were obtained during the interview and the products sourced later from the market. The component of the herbal products was obtained from the product label. The native names of the plant species in the crude herbal medicines was used to identify their full botanical names from African Herbal Pharmacopoeia [34]. Where only the English names of the plant species in the refined packaged herbal medicines were provided on the product label, the botanical names were sourced from African Herbal Pharmacopoeia [34] and online from The International Plant Names Index [35].

### Data analysis

Data were analysed using SPSS 17. Results were presented as median with inter-quartile range (IQR) for time related variables and as frequencies and percentages for other variables. The relationship between status of herbal medicine use and level of education or type of occupation of the respondents were determined by using a Pearson's chi-square at *P *< 0.05 significant level.

## Results

The highest number of the respondents (186; 47.9%) was in the age range 21-30 years (median = 43.4 years, inter-quartile range = 24-52 years) and are male preponderant (M: F, 2.3:1). They were single (240; 61.9%), married (147; 37.9%) or divorced (0.3%). The majority of the respondents (182; 46.9%) was either in or had completed secondary school education. Similarly, 178 (45.9%) respondents had attained a tertiary education; 11 (2.8%) had only primary education and the rest of them never had any formal education. With regards to their occupation, 143 (36.9%) respondents were unskilled workers; 137 (35.3%) were either students or unemployed; 108 (27.9%) were skilled workers.

Over half of the respondents (259; 66.8%) were herbal medicine users (the type of herbal medicines used ranged from crude forms, packaged herbal products to dietary or nutritional supplement); 129 (33.2%) respondents were non- users of herbal medicine; and none of them were herbal medicine-exposed.

Herbal medicines were used for various purposes indicated in Table 1. They were used most frequently for malaria (54; 20.8%) and for reducing blood sugar level (42; 16.2%). Herbal medicine non-users avoided the preparation or product because they were ineffective (0.8%); bitter to taste (2.3%); personally, they disliked herbal medicines (23; 17.8%); lack of faith in herbal medicines (28; 21.7%); and herbal medicines were likely unsafe (34; 26.4%). Forty (31%) herbal medicine non-users gave no reason for avoiding herbal medicine preparations or products.

**Table 1:** The reasons for using herbal medicines by the respondents

Reasons for using herbal medicine	Number of herbal medicine users	Percentage (%)
No specific reason	67	25.9

Malaria	54	20.8

Blood sugar level reduction	42	16.2

Fever	26	10.0

Teeth cleaning	16	6.2

General body pain relief	12	4.6

Diarrhoea	9	3.5

Fatigue	9	3.5

Menstrual pain	4	1.5

Self protection	4	1.5

Skincare	3	1.2

General illness	3	1.2

Dysentery	3	1.2

Strength gain	2	0.8

Blood cleansing	2	0.8

Cholera	2	0.8

Blood enrichment	1	0.4

The specific herbal medicine preparations/products used by the respondents are listed in Table 2.

**Table 2:** Pattern of herbal medicines used by the respondents

Name of herbal medicine preparation	Components	Frequency (%) (N = 198)
'Agbo jedi-jedi'	Scented-leaves (*Pelargonium zonale *(L.) L'Hér.), grapefruit (*Citrus paradisi *Macfad.) juice extracts, bitter leaf (*Vernonia amygdalina *Delile), Sorghum (*Sorghum bicolour *Moench) leaves, naphthalene tablets, garlic (*Allium sativum *L.)	69 (35.0%)

'Agbo iba'	Bark of pineapple (*Ananas comosus *(L.) Merr.) fruit, paw paw *(Carica papaya *L.) leaves and seeds, 'Dongoyaro' (*Azadirachta indica *A. Juss.) leaves, lime juice, lemon grass (*Cymbopogon citrates *Stapf.) leaves, guava (*Psidium guajava *L.) leaves, scented- leaves (*Pelargonium zonale *(L.) L'Hér.)	54 (27.5%)

Oroki herbal mixture^® ^	Stem bark of African mahogany (*Khaya ivorensis *A.Chev.) tree, pattern wood (*Alstonia congensis *Engl.), mango (*Mangifera indica *L.) leaves, Sorghum (*Sorghum bicolour *Moench)	18 (9.0%)

Herbal tooth paste	Aloe vera (*Aloe barbadensis *Mill.)	15 (7.5%)

Ajase poki-poki^® ^	Tobacco (*Nicotiana *L.) leaves, stem bark of coconut (*Cocos nucifera *L.), seeds and coat of alligator pepper (*Aframomum melegueta *K.Schum.)	10 (5.0%)

Yoyo bitter^® ^	Bitter leaf (*Vernonia amygdalina *Delile), ginger (*Zingiber officinale *Roscoe), scented- leaves (*Pelargonium zonale *(L.) L'Hér.)	8 (4.0%)

'Ijebu-ode' mixture drink	Mushroom (*Ganoderma lucidum*), Coconut (*Cocos nucifera *L.) oil and roots	7 (3.5%)

Splina	Splina (*Bucataria corpului*), natural honey	7 (3.5%)

Omega root	Coconut (*Cocos nucifera *L.) oil	5 (2.5%)

Jobelyn^® ^	Sorghum (*Sorghum bicolour *Moench) leaves	4 (2.0%)

Dudu-Osun soap^® ^	Palm kernel (*Elaeis guineensis *A. Chev.) oil	4 (2.0%)

Alomo bitter^® ^	African breadfruit (*Treculia Africana *Decne. Ex Trécul), stem bark of African mahogany (*Khaya ivorensis *A. Chev.)	2 (1.0%)

Twelve different types of herbal medicine preparations and products (either alone or in combination with other herbal medicines) were used by the respondents. 'Agbo jedi- jedi' (91; 35.1%) and 'agbo iba' (71; 27.4%) preparations were used most frequently.

Friends, relatives and colleagues influenced 117 (45.2%) of the respondents to use herbal medicine. The respondents' other sources of information about herbal medicine included their parents (77; 29.7%); health professionals (13; 13%); herbal medicine retailers (11; 4.2%); media: television, radio and newspaper advertisements (3.5%); their spouse (3.5%); and herbal medicine practitioners (0.8%). Seventeen (6.6%) herbal medicine users did not disclose their source of information about herbal medicines.

The use of herbal medicine, based on the respondents' level of education, is presented in Table 3.

**Table 3:** Relationship between the educational levels and herbal medicine use status of the respondents

Educational level	Herbal medicine use status		Total
	Non users	Users	
None	4 (23.5%)	13 (76.5%)	17 (100.0%)

Primary	7 (63.6%)	4 (36.4%)	11 (100.0%)

Secondary	53 (29.1%)	129 (70.9%)	182 (100.0%)

Tertiary	65 (36.5%)	113 (63.5%)	178 (100.0%)

**Total**	**129 (33.2%)**	**259 (66.8%)**	**388 (100.0%)**

χ^2 ^= 7.55, p = 0.056 (no significant difference)

There was no statistically significant difference between the status of herbal medicine use and respondents' levels of education (χ^2 ^= 7.55, p = 0.056). Table 4 shows that status of herbal medicine use was significantly associated with the respondents' occupation (χ^2 ^= 32.8, p = 0.000).

**Table 4:** Relationship between the profession and herbal medicine use status of the respondents

Occupation	Herbal medicine use status		Total
	**Non users**	**Users**	

Professionals	19 (39.6%)	29 (60.4%)	48 (100.0%)

Skilled	7 (11.7%)	53 (88.3%)	60 (100.0%)

Students/Unemployed	67 (48.9%)	70 (51.1%)	137 (100.0%)

Unskilled	36 (25.2%)	107 (74.8%)	143 (100.0%)

**Total**	**129 (33.2%)**	**259 (66.8%)**	**388 (100.0%)**

χ^2 ^= 32.8, p = 0.000 (significant difference)

Over half (58%) of the herbal medicine users considered herbal medicines safe to use; 89 (22.9%) believed otherwise; and 74 (19.1%) were uncertain. Safety of herbal medicines were attributed to their natural origin [88/150 (58.7%)]; efficacy [51/150 (34%)]; and lack of adverse effects [11/150 (7.3%)]. Among the respondents who considered herbal medicine unsafe, non-pharmaceutical preparation and non-packaging of the products [27/89 (30.3%)] were the only reasons alluded.

The respondents found herbal medicines effective (159; 41%); ineffective (123; 31.7%) or indeterminate (106; 27.3%). A high proportion (79.2%) of herbal medicine users believed that herbal medicines have no adverse effects. The rest of them (20.8%) had experienced one or more adverse effects following the use of herbal medicines which included skin rashes (7; 13%); vomiting (7; 13%); dizziness (6; 11.1%); frequent stooling (3; 6%); and abdominal pain (3; 6%).

Twenty eight (52%) herbal medicine users experienced inexplicable adverse effects following the use of herbal medicines.

## Discussion

The use of herbal medicines has been extensively studied in Nigeria among adult and paediatric population with chronic illnesses such as epilepsy [26], hypertension [27], diabetes mellitus [28], cancer [33], sickle cell anaemia and asthma [30]. Only a very few studies have specifically evaluated herbal medicine use among the general population [36,37]. The current study assessed the prevalence of herbal medicine use among a general population of adults without chronic illnesses. A high prevalence of 66.8% observed in our study is similar to the rate (69.4%) observed in another adult population (with or without chronic illnesses) in Nigeria where herbal medicines were used concurrently with conventional medicines [32]. However, the current rate of herbal medicine use was higher than the rates reported in Nigeria among adults with hypertension (39.1%) [27], diabetes mellitus (46%) [28], epilepsy (47.6%) [26] and cancer (51.9%) [33], and similarly higher than the rates (37.8%- 40%) reported among adult patients in a setting of a health maintenance organization in Central Texas city, United States of America [36] and among a general population in Finland, where herbal remedies were defined in the context of alternative medicines [38]. The current rate was also almost thrice the value (23%) reported in children with asthma, epilepsy and sickle cell anaemia [30].

Herbal medicines were used for a variety of health conditions ranging from malaria to blood enrichment (Table 1). Contrasting findings have been reported in the United States where herbal remedies were predominantly used to treat common cold and for general health maintenance [36]. Malaria was the commonest indication for herbal medicine use in this study similar to a previous study in Nigeria [32]. However, only 20% of our population used herbal medicines to treat malaria compare to 80% in the previous study [32]. The wide disparity in the proportions of herbal medicine users for malaria in both studies may be as a result of the differences in herbal medicine definition. While we define herbal medicines as the use of plants' parts for medicinal purposes, other studies defined herbal medicines as finished, labeled medicinal products of plant or non-plant origin. Malaria is a common public health problem in Nigeria that may adversely affect both human and capital resources. The emergence of chloroquine and sulphadoxime/pyrimethamine resistant malaria in Nigeria may have informed the use of herbal medicines by the respondents.

It is of concern that one-fifth of herbal medicine users in our study had no specific reason for the use. The lack of knowledge of potential harms of herbal medicines may have encouraged this practice. Given the high proportion of the participants (66.8%) who were herbal medicine users and the wide range of indications for their use (Table 1), it is remarkable that only 12 herbal medicine preparations, involving 22 plants species, were used by the respondents. This finding is however contrasting to other studies that reported the use of high number of different plant species for chronic diseases such as diabetes in South Africa [39], inflammatory diseases in South-western Nigeria [40], and a wide range of acute and chronic illnesses in India [41]. The exclusion of participants with chronic illnesses may have accounted for the low use of herbal medicine preparations observed in our study. The range of herbal medicines used by the respondents is quite different from those reported in other studies in Nigeria [26-30,32,33], Finland [38] and the United States [36]. This may be explained by the varied health conditions and cultural differences in each of the populations studied. Of the 12 different herbal medicine preparations used by the respondents, four of them ('agbo jedi jedi', 'agbo iba', 'ijebu ode' mixture and splina) were in their crude forms, while the remaining eight were refined into packaged forms. The regulatory framework in Nigeria has encouraged local production of herbal medicines in refined packaged forms [42], hence the proliferation of packaged herbal medicines in the Nigerian markets. Only the eight packaged herbal medicine preparations are likely to be certified by the National Agency for Food Drug Administration and Control (NAFDAC) for human use which may guaranty their safety [43]. However, many of the herbal medicine preparations may supposedly contain similar ethnobotanical plant species (Table 2), thus may put the respondents practicing herbal medicine polypharmacy at risk of toxicity. Adequate labeling of herbal medicine preparations and their packaged products with the constituent elements and a general public enlightenment programme on the need to read herbal medicine product labels very well may be necessary to avert herbal medicine toxicity. The levels of education of herbal medicine users have been shown to significantly influence their use in Nigeria [32] but contrarily, in this study, there was no significant association between herbal medicine use and users' level of education. The influence of relatives, friends and neighbours on health-care seeking preferences for herbal medicines has been reported globally in both adults and children [26,30,36]. The high percentage (78.4%) of the respondents in this study influenced by parents, relatives, spouses, friends and colleagues to use herbal medicine preparation or products is comparable to the 60%-86% previously reported in Nigeria [26,30,44] but higher than the 51.4% reported in the United States [36]. Nevertheless, these findings further corroborate the fact that knowledge of herbal medicines are handed down from parents, relatives and friends and may not necessarily require any formal education [3,45].

Herbal medicines were considered safe by over half of the users. Safety of herbal medicines was erroneously attributed to their natural sources. This misconception was one of the reasons for using herbal medicines by pregnant women in Nigeria [29] and other people in the developed countries [46,47]. The fact that herbs are of natural origin does not automatically guaranty their safety [47]. Several cases have been reported of herbal medicine preparations or products being adulterated with heavy metals [21,48], orthodox medicines [49,50] or contaminated with microbes [51,52]. The potential toxicity of herbal medicine preparations or products [22], as well as lack of safety warnings on their labels, [23] are of concern regarding their use.

The majority of the respondents who were herbal medicine users erroneously believed that adverse effects rarely occur with its use. This confirms the findings from previous studies [29,42,43]. Although, a few respondents experienced some adverse effects; they were neither severe nor life threatening. Previous studies have, however, associated severe acute renal failure [23] and hepatic failure [24] to the use of herbal medicines. The proportion of the respondents (20%) who experienced mild to moderate adverse effects is considerable and much higher than the proportion (10.4%) who reported side effects to herbal remedies in the United States [36]. Further studies are therefore necessary in the future to assess the specific potential toxicities associated with different herbal remedies.

One of the limitations of this study is that concurrent use of orthodox with herbal medicines was not evaluated. This is because individuals with chronic illnesses, who are likely to use prescribed orthodox medicines regularly, were excluded from the study. Our data represent one point in time and do not reflect changes in patients' experiences with herbal medicines over time. Although, the demographics of the herbal medicine users in this study were similar to the characteristics of users previously reported [32,36], our findings may not be necessarily generalizable to other populations in Nigeria.

## Conclusions

Herbal medicine use among adults without chronic illnesses is quite high in Lagos. Many of the respondents found herbal medicines to be safe, effective and beneficial. Despite the belief of many of the respondents that herbal medicines rarely produce adverse effects, a few experienced them mildly and moderately. Considering the magnitude of popularity of herbal medicines among the respondents and their levels of ignorance of the potential toxicities, it is necessary to evaluate the safety, efficacy and quality of these preparations and products which may involve clinical trial studies. Public enlightenment programme, in the form of health education about safe use of herbal medicines, may be a useful means of minimizing the potential adverse effects.

## List of abbreviations

TBA: Traditional Birth Attendant; WHO: World Health Organization; LGA: Local Government Area; CAM: Complementary and Alternative Medicine; HIV-Human Immunodeficiency Virus; AIDS: Acquired Immunodeficiency Syndrome; IQR: Interquartile Range; ACT: Artemesinin Combination Therapy; NAFDAC: National Agency for Food Drug Administration and Control.

## Competing interests

The authors declare that they have no competing interests.

## Authors' contributions

IAO conceived and participated in the design of the study, reviewed the results, and critically reviewed the manuscript. KAO participated in the study design, analysed the results, and contributed to drafting the manuscript. MA participated in the study design, interviewed the participants, performed the data entry, and critically reviewed the manuscript. All authors read and approved the final manuscript.

## Pre-publication history

The pre-publication history for this paper can be accessed here:

http://www.biomedcentral.com/1472-6882/11/117/prepub
